# Preparation, Cell Compatibility and Degradability of Collagen-Modified Poly(lactic acid)

**DOI:** 10.3390/molecules20010595

**Published:** 2015-01-05

**Authors:** Miaomiao Cui, Leili Liu, Ning Guo, Ruixia Su, Feng Ma

**Affiliations:** School of Chemical & Pharmaceutical Engineering, Qilu University of Technology, 3501 Daxue Road, Jinan 250353, China; E-Mails: qljn113@yeah.net (M.C.); hgzy88@yeah.net (N.G.); jinanbaojie@sina.com (R.S.); fm8077@sina.com (F.M.)

**Keywords:** poly(lactic acid), collagen, hydrophilicity, cell compatibility, degradability

## Abstract

Poly(lactic acid) (PLA) was modified using collagen through a grafting method to improve its biocompatibility and degradability. The carboxylic group at the open end of PLA was transferred into the reactive acylchlorided group by a reaction with phosphorus pentachloride. Then, collagen-modified PLA (collagen-PLA) was prepared by the reaction between the reactive acylchlorided group and amino/hydroxyl groups on collagen. Subsequently, the structure of collagen-PLA was confirmed by Fourier transform infrared spectroscopy, fluorescein isothiocyanate-labeled fluorescence spectroscopy, X-ray photoelectron spectroscopy, and DSC analyses. Finally, some properties of collagen-PLA, such as hydrophilicity, cell compatibility and degradability were characterized. Results showed that collagen had been grafted onto the PLA with 5% graft ratio. Water contact angle and water absorption behavior tests indicated that the hydrophilicity of collagen-PLA was significantly higher than that of PLA. The cell compatibility of collagen-PLA with mouse embryonic fibroblasts (3T3) was also significantly better than PLA in terms of cell morphology and cell proliferation, and the degradability of PLA was also improved after introducing collagen. Results suggested that collagen-PLA was a promising candidate for biomedical applications.

## 1. Introduction

Poly(lactic acid) (PLA) is receiving considerable attention in biomedicine and environmental applications because of its excellent biodegradability, mechanical properties, and biocompatibility. The principal raw material of PLA is lactic acid, which is nonirritating and safe for human use. The degradation products of PLA are CO_2_ and H_2_O, which are nontoxic. Thus, PLA has been widely used in biomedical materials, such as resorbable medical sutures [1], dental materials [2,3,4], ophthalmic implant materials [5,6,7], fracture fixation devices [8,9], tissue engineering [10,11,12,13], and drug delivery systems [14,15,16,17,18]. However, PLA is highly hydrophobic, which results in low cell affinity. Moreover, it elicits an inflammatory response from the living host upon direct contact with biological fluids [19]. Therefore, the hydrophilic and hydrophobic balance of PLA should be controlled. Moreover, the properties of PLA, such as biocompatibility and degradability, should be improved.

Many methods have been developed to improve the properties of PLA, which contain block copolymerization and graft copolymerization. Compared with block copolymerization, graft modification improves PLA the properties with less influence on the main chain. Some methods in prior literature involve graft modification, which include UV-photo grafting [20,21,22] and covalent linking of hydrophilic polymers [23,24] on PLA. Mario H. *et al.* [21] introduced N-vinylpyrrolidone (NVP) onto PLA film through photoinitiated grafting to modify the nature hydrophobic PLA behavior. The carboxylic groups on macromolecules are often used to produce a covalent linking reaction. Gao H. *et al.* [25] synthesized PLA grafted cyclodextrin by a direct reaction of the carboxylic group on PLA with an amino group on aminolyzed cyclodextrin using dicyclohexylcarbodiimide as the catalyst. The carboxylic groups on macromolecules can also be transferred into a more active acylchlorided group for a covalent linking reaction. Chen X.Q. *et al.* [26] transferred a carboxylic group on carboxymethyl-β-cyclodextrin to an acylchlorided group. Then, β-cyclodextrin was grafted onto chitosan by the reaction of acidamide.

Introducing natural biomacromolecules, such as cellulose [27], dextran [28], chitosan [29], cyclodextrin derivatives [30], and chondroitin sulfate [31], into PLA through graft modification is one approach to improving the biocompatibility of PLA. Collagen is one of the main components of the extracellular matrix in animal bodies. The excellent biocompatibility and safety because of its biological characteristics, such as biodegradability and weak antigenecity, makes collagen suitable for cell adhesion and cell proliferation [32,33,34]. The introduction of collagen into PLA can enhance the hydrophilicity and biocompatibility. Hong *et al.* [23] fabricated CPLA microspheres by introducing collagen onto the surface of PLA microspheres through aminolysis and grafting-coating. Li *et al.* [35] grafted collagen on both ends of PLA using dicyclohexylcarbodiimide (DCC) as a condensing agent.

**Scheme 1:**
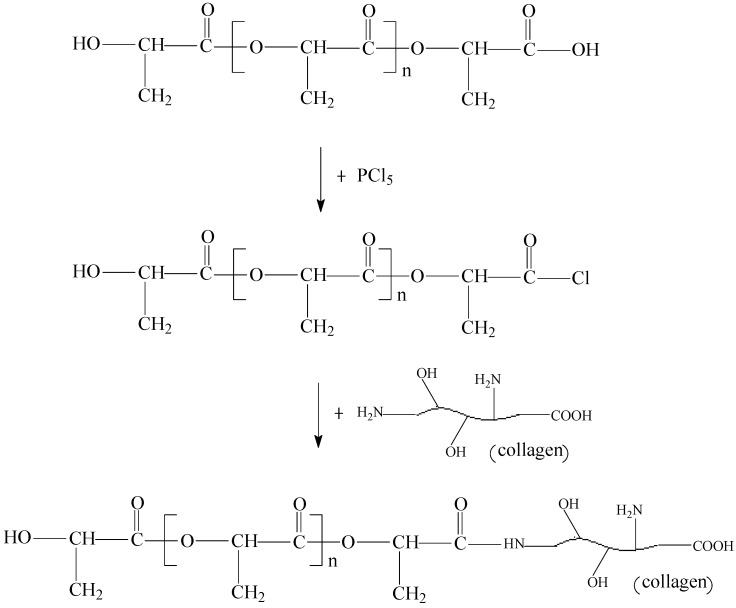
Schematic of collagen-poly(lactic acid) (PLA) synthesis.

In this study, we prepared the collagen-modified poly(lactic acid) (collagen-PLA) by using a grafting method (Scheme 1). The carboxylic group at the open end of PLA was transferred into the reactive acylchlorided group through a reaction with phosphorus pentachloride. Then, collagen-modified PLA was synthesized through the reaction between the reactive acylchlorided group and amino or hydroxyl groups on collagen. Subsequently, the structure of collagen-PLA was confirmed by using Fourier transform infrared (FTIR) spectroscopy, fluorescein isothiocyanate (FITC)-labeled fluorescence spectroscopy, X-ray photoelectron spectroscopy (XPS), and DSC thermal analyses. Finally, some properties of collagen-PLA, such as hydrophilicity, cell compatibility and degradability were characterized.

## 2. Results and Discussion

### 2.1. Preparation of Collagen-PLA

**Figure 1:**
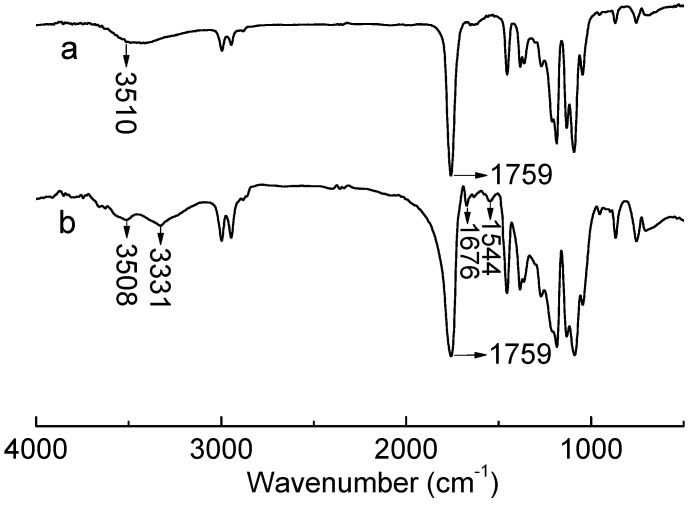
FTIR spectra of (a) PLA and (b) collagen-PLA.

**Figure 2:**
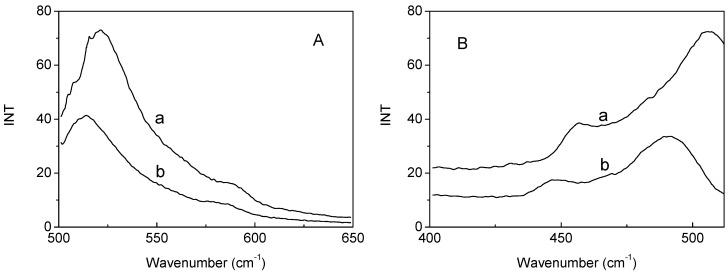
Fluorescence (**A**) emission spectra and (**B**) excitation spectra of (a) FITC-labeled collagen-PLA and (b) collagen.

**Figure 3:**
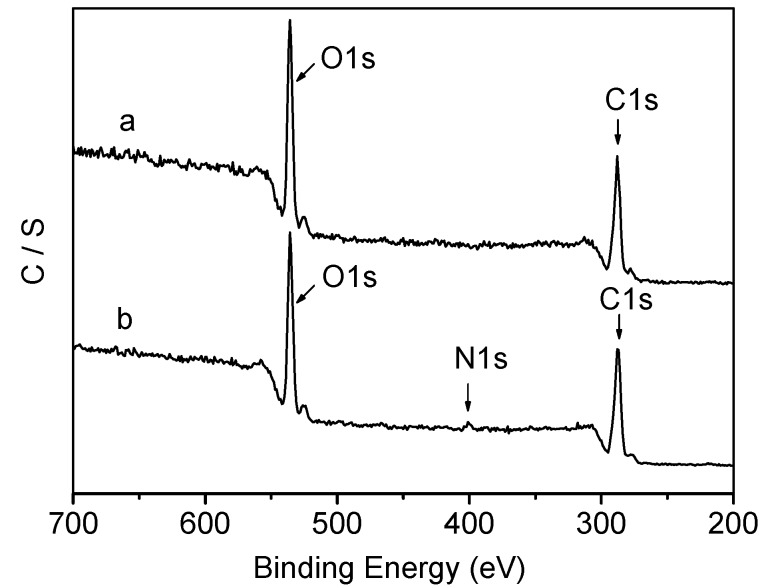
XPS spectra of (a) PLA and (b) collagen-PLA.

The prepared collagen-PLA was characterized using FTIR (Figure 1), FITC-labeled fluorescence spectra (Figure 2), and XPS (Figure 3). The FTIR spectra of PLA and collagen-PLA are depicted in Figure 1a,b, respectively. Figure 1a shows that the wide and weak peak at 3510 cm^−1^ was because of the vibration of O-H in the hydroxyl group and carboxyl groups as it stretched. The sharp peak at 1759 cm^−1^ was attributed to the vibration of C=O in carboxyl groups as it stretched. Figure 1b shows that the peak of stretching vibration at 3329 cm^−1^ was assigned to O-H, NH_2_, and N-H in the amide group, which was not found in FTIR spectrum of PLA. The C=O peak at 1759 cm^−1^ was wider than that of PLA. The peaks at 1671 and 1526 cm^−1^ were ascribed to the stretching vibration of C=O and bending vibration of N-H in amide group, which was also not found in the FTIR spectrum of PLA. Thus, collagen had been grafted onto the PLA.

In this study, FITC was employed to label the obtained collagen-PLA polymer. FITC can react with collagen under alkaline conditions, which result in maximum excitation spectra in the region of 450–500 nm and the maximum emission spectra in the region of 500–530 nm. Figure 2 shows that hydrolyzed collagen and collagen-PLA had obvious emission and excitation spectra in the respective regions. Thus, collagen had been covalently incorporated into PLA through the grafting method.

The XPS spectra of PLA and collagen-PLA are shown in Figure 3. Two peaks in XPS spectra of PLA, corresponded to C1s (binding energy = 287 eV) and O1s (binding energy = 534 eV), respectively. 4b shows that the 402 eV peak was assigned to N1s, which was not found in the XPS spectra of PLA. Element N come from the collagen grafted onto PLA. Thus, collagen had been grafted onto PLA.

### 2.2. Thermal Properties

**Figure 4:**
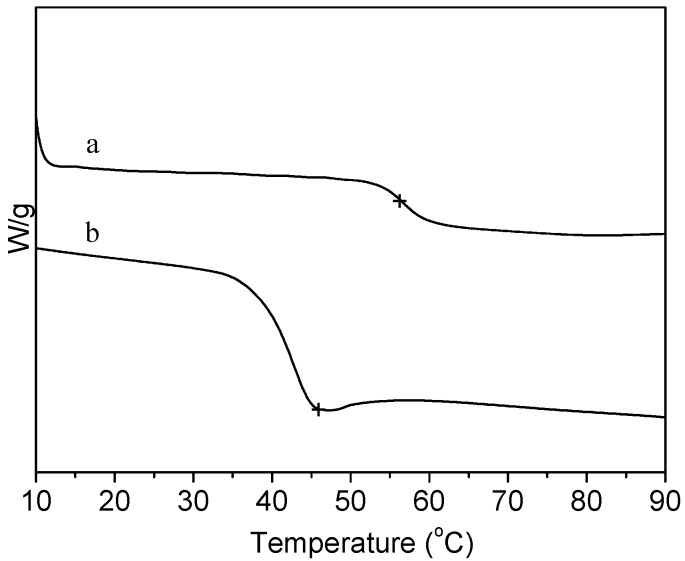
DSC curves of (a) PLA and (b) collagen-PLA.

The DSC curves of PLA and collagen-PLA are showed in Figure 4. The glass-transition temperature (*T*_g_) of PLA was 56.0 °C, whereas the *T*_g_ of collagen-PLA was 46.0 °C, which differed from that of PLA and collagen (approximately 43 °C). Thus, collagen was grafted onto PLA instead of being mixed with PLA.

### 2.3. Graft Ratio of Collagen and Relative Molecular Mass

Ninhydrin can react with collagen to generate blue-violet condensation compound that has a maximum absorption peak at 580 nm. Therefore, we can make use of the ninhydrin reaction for the quantitative determination of collagen content on collagen-PLA. The graft ratio was measured to be approximately 5%.

In this study, the relative molecular mass was measured using a gel permeation chromatograph. The relative molecular mass (*M*_w_) of PLA and collagen-PLA was 10,053 (PDI = 2.309) and 11,638 (PDI = 2.925), respectively. Thus, the *M*_w_ of collagen-PLA was slightly higher than that of PLA because of the collagen that was introduced to PLA.

### 2.4. Hydrophilicity

Water contact angle of material is associated with its hydrophilicity. In general, the smaller the water contact angle, the higher the hydrophilicity. The water contact angle of PLA and collagen-PLA were approximately 72° and 56°, respectively. Thus, the hydrophilicity of collagen-PLA is higher than that of PLA. PLA could introduce some hydrophilic groups, such as -NH_2_, -COOH and -OH, and enhance the hydrophilicity of copolymers when collagen is grafted on PLA.

**Figure 5:**
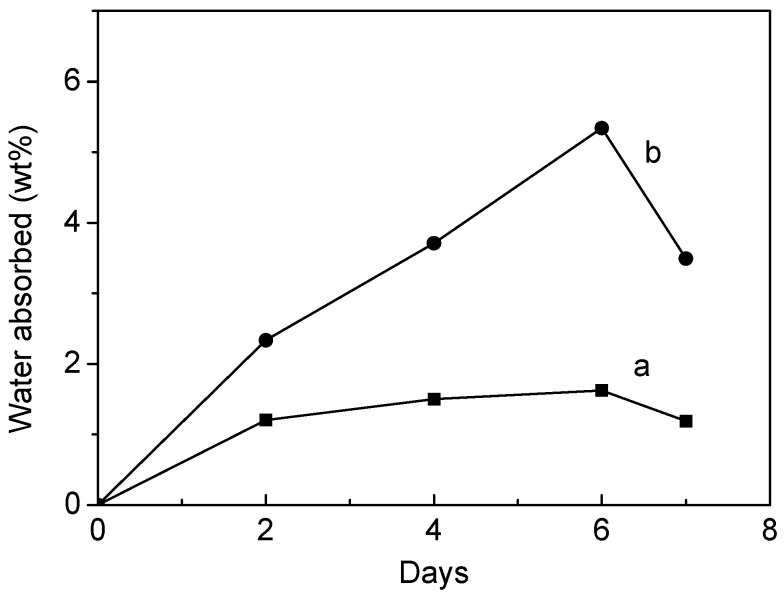
Water absorption behavior of (a) PLA and (b) collagen-PLA.

The water absorption illustrated the balance between the dissolution of oligomers and water uptake of the residual material [36]. Water absorption behaviors shown in Figure 5 indicate that the water absorption of collagen-PLA was significantly higher than PLA and exhibited a downward trend after 6 days. Collagen-PLA had more hydrophilic groups, such as -NH_2_, -COOH, and -OH, than PLA. These groups could increase water absorption and degrade copolymers. After 6 days, the dissolution of oligomers predominated in the system, which contributed to the decline of water absorption.

### 2.5. Cell Compatibility

**Figure 6:**
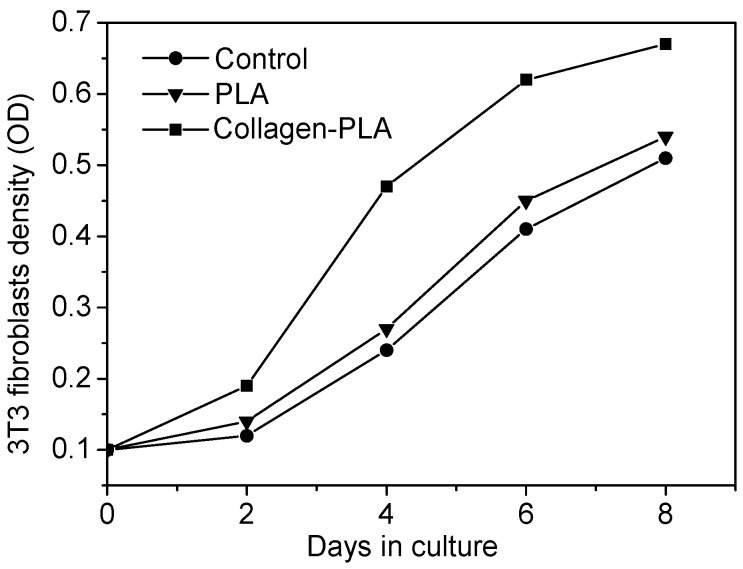
Cell growth curves of 3T3 fibroblasts on PLA and collagen-PLA with glass as control.

Figure 6 shows the optical micrographs of 3T3 fibroblasts cultured on glass as control, PLA, and collagen-PLA on the sixth day of culture. The morphology observations revealed that 3T3 fibroblasts adhered and spread on the surfaces of glass and samples films. The cell morphology presented spindle shapes and triangle shapes after 24 h of culture. After 6 days of culture, 3T3 cells cultured on different samples surfaces took on a different cell morphology and cell density. The 3T3 cells cultured on PLA film surface still presented spindle-shaped and triangle shape, and some cells contact, aggregated together and unevenly spread on PLA film surface, with a higher cell density than that of control. After 6 days of culture, 3T3 cells cultured on the collagen-PLA film surface were shaped like round strips, and most cells came into contact, aggregated, and spread uniformly around the collagen-PLA surface. They came into contact and aggregated more closely. They nearly formed enough cells to cover the substrate surface. Moreover, the cell density on collagen-PLA surface was clearly higher than that of PLA and glass.

**Figure 7:**
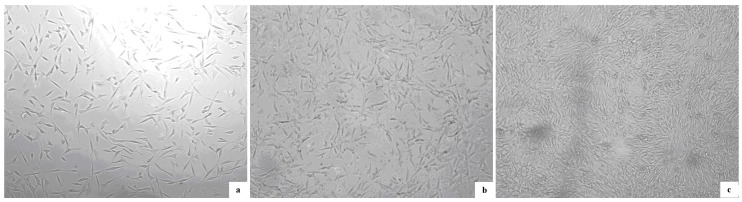
Optical micrographs (100×) of 3T3 fibroblasts cultured on (**a**) glass as control; (**b**) PLA; and (**c**) collagen-PLA.

The proliferation of 3T3 fibroblasts on the samples surfaces was presented in Figure 7. The proliferation trend of 3T3 cells on all test groups was almost similar. The growth rate of 3T3 cells on the collagen-PLA surface was obviously higher than that of PLA and glass. On the 4th day of culture, the growth rate of 3T3 on the collagen-PLA surface was two times that on the PLA surface and 2.8 times that on glass, which indicates that the growth rate and vigor of 3T3 cells on collagen-PLA surface was the best. These results showed that the cell compatibility of collagen-PLA with mouse embryonic fibroblasts (3T3) was significantly improved compared with PLA.

### 2.6. Degradability

**Figure 8:**
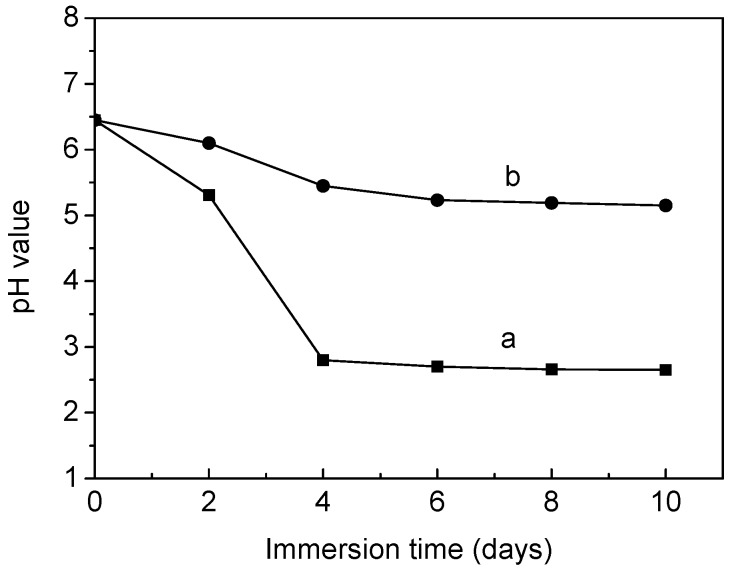
The pH change of medium as a function of time during degradation of (a) PLA and (b) collagen-PLA.

The medium pH change of PLA and collagen-PLA *versus* degradation time are shown in Figure 8. On the first 2 days, the medium pH of PLA changed from 6.45–5.25. Then, it dropped to 2.80 on the fourth day. Then, the curve of pH *versus* degradation time remained essentially constant. These results indicated that the medium showed strong acidity as PLA degraded. However, the pH of collagen-PLA changed from 6.45–5.15 in 10 days. Thus, the medium was slightly acidic as collagen-PLA degraded. Moreover, the acidity of the medium was less than that of PLA. The medium pH was mainly influenced by the release of -COOH, which was produced in the hydrolysis process of the ester bond in the main polymer chain. Many -NH_2_ groups on the introduced collagen can neutralize H^+^ generated through the ionization of -COOH. Thus, the medium acidity of collagen-PLA was obviously less than that of PLA.

**Figure 9:**
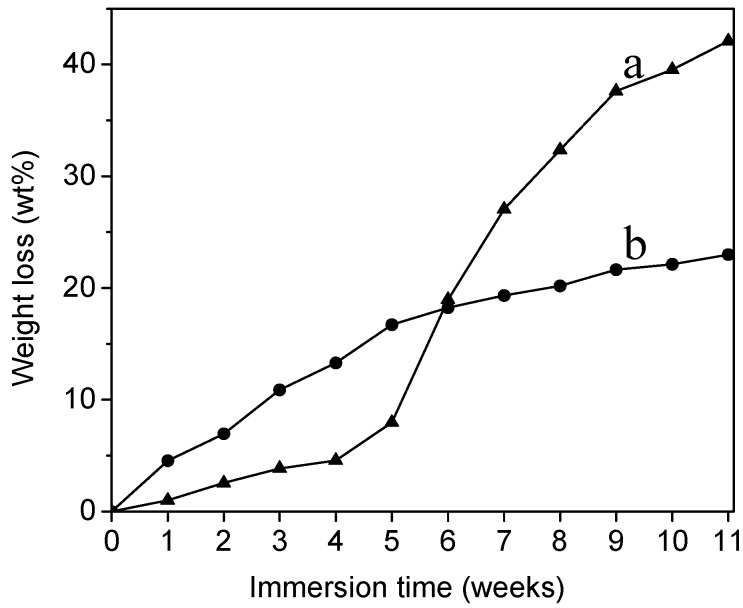
Weight loss of (a) PLA and (b) collagen-PLA *versus* degradation time.

Figure 9 shows the weight loss of PLA and collagen-PLA *vs.* degradation time. The degradation trend of PLA and collagen-PLA was basically the same during the first four weeks, and the weight loss of collagen-PLA was higher than PLA in the first six weeks, mainly because of the increased hydrophilicity of collagen-PLA. However, from the fourth week, the weight loss of PLA increased at an abnormally rapid rate while the weight loss of modified PLA continued to increase at a relatively constant rate; the weight loss of PLA become significantly higher than collagen-PLA, which was probably attributed to the acid-catalyzed self-accelerating degradation of PLA [35]. Figure 8 shows the medium showed strongly acidic during degradation of PLA, which result in acid-catalyzed degradation and then accelerated PLA degradation. This phenomenon showed that the degradability of PLA had been improved by grafting collagen. In addition, the degradation rate was more constant, which is very important for biomedical materials.

## 3. Experimental Section

### 3.1. Materials

Lactic acid (AR) and Phosphorus pentachloride (PCl_5_) were purchased from the Shanghai Chemical Reagent Company, China. Collagen (*M*_w_ = 600–3000) was supplied by Shandong Qingzhou Longbei biotechnology Co., Ltd., Qingzhou, China. Ninhydrin and FITC (HPLC grade) were purchased from Sigma Company, St. Louis, MO, USA. Mouse embryonic fibroblasts (3T3) were supplied by the Shandong Medicinal Biotechnology Centre, Jinan, China. The α-MEM medium was purchased from Gibco Company, Grand Island, NY, USA. Fetal bovine serum was supplied by Hangzhou Sijiqing Company, Hangzhou, China. CCK-8 was purchased from Dojindo Molecular Technologies, Inc., Mashikimachi, Kamimashiki gun Kumamoto, Japan. All reagents were used as received without further purification.

### 3.2. Preparation of Collagen-PLA

Polylactide was directly synthesized through melt polycondensation using stannous chloride (SnCl_2_, AR) as a catalyst [37,38]. The PLA was dissolved in dichloromethane at 35 °C, to which PCl_5_ was protected using N_2_. Then, the solution was stirred for 1 h and protected using refluxing. Subsequently, the dichloromethane was removed, and the precipitate was dissolved in dimethyl sulfoxide (DMSO, AR), to which the solution of collagen in DMSO was added. The solution was stirred at 50 °C for 8 h. Then, the reaction solution was added to distilled water to precipitate collagen-PLA. The solution was filtered and washed repeatedly until the filtrate could not be colored with ninhydrin. Finally, the collagen-PLA was dried in vacuum at 37 °C for 48 h.

### 3.3. Characterization

FTIR spectra were obtained using Fourier transform infrared spectrometer (IRPrestige-21, Shimadzu, Kyoto, Japan). Fluorescence spectra were obtained through the fluorescence labeling method using a fluorescence spectrometer (F-4500, Hitachi, Tokyo, Japan) [39]. XPS spectra were measured through XPS (PHI5300, PE-PHI, Waltham, Massachusetts, USA). DSC thermal analysis was performed with differential scanning calorimeter (TAQ10, TA, New Castle, DE, USA) under an atmosphere of N_2_ (at a rate 20 mL/min) at a heating rate of 15 °C/min and scanned from 0–200 °C. The relative molecular mass (Wight-average molecular weight, *M_w_*) of PLA and collagen-PLA was measured with gel permeation chromatograph (SP-4270, OI, College Station, TX, USA). The graft ratio of collagen on PLA was determined through a ninhydrin reaction [40].

### 3.4. Hydrophilicity and Degradability Test

The hydrophilicity test was carried out through measuring water contact angle and water absorption. Water contact angle was determined using a contact angle/interface tension measuring instrument (JC2000C1, POWEREACH, Shanghai, China). Water absorption was calculated as the percentage ratio of the amount of water absorbed to the initial dry weight.

Degradability test was performed by measuring the pH of distilled water and weight loss of samples. The former was measured by submerging samples in distilled water (pH 6.45) at 37.0 ± 1 °C. The pH of medium was surveyed at 2-day intervals. The latter was measured by submerging samples in phosphate buffer salines (PBS) (pH 7.4, 0.1 M) at 37.0 ± 1 °C. Samples were removed, dried at 40 °C for 24 h, and weighted at 1 week intervals. The weight loss ratio was calculated according to the equation:

(1) $$\mathrm{Weight loss ratio}=\frac{W_{1}-W_{2}}{W_{1}}\times 100\%$$

where *W*_1_ and *W*_2_ are weights of the sample before and after the hydrolytic degradation, respectively.

### 3.5. Cell Compatibility Characterization

In this paper, the cell compatibility of collagen-PLA with mouse embryonic fibroblasts (3T3) was studied in the respects of cell morphology and cell proliferation. Test groups were divided into three groups: control, PLA and collagen-PLA group. Each group had five samples for repeated test. About 0.5 g of PLA and 0.5 g of collagen-PLA with a concentration of 50 g/L were dissolved in acetic ether and tetrahydrofuran, respectively. Then, 0.2 mL of these solutions was added to glass culture bottles separately, to which another 0.2 mL of solvent was added, and then evaporated at room temperature for 48 h to form sample films at the bottom of the culture bottle uniform. In the culture bottles of the control group, 0.2 mL of solvent was added and evaporated at room temperature for 48 h. All bottles were sterilized through ultraviolet ray light for 0.5 h before use. Mouse embryonic fibroblasts (3T3) were resuscitated. Then, 3T3 were incubated in the culture medium (α-MEM medium) supplemented with 10% (v/v) fetal bovine serum and 0.35 g/L glutamine in incubator at 37 °C with 5% CO_2_ in air. The culture medium was changed once every 2 days. After 4 or 5 days, the 3T3 cells passage and the cells of the second generation were harvested for later use.

The 3T3 cells were plated at a cell density of 1 × 10^5^/bottle at the bottom of the culture bottle that contains the sample films. On the 6th day of culture, the cell growth was investigated under a Leica inverted fluorescence microscope.

The 3T3 cells were plated in 24 glass culture bottles (volume = 25 mL): 12 glass culture bottles for the PLA group and 12 glass culture bottles for the collagen-PLA group. After 2, 4, 6, and 8 days of culture, cell proliferation on samples was measured using the CCK-8 method. The optical density value was read at the wavelength of 450 nm using a microplate reader (MK3, Labsystems, Helsinki, Finland).

## 4. Conclusions

The collagen-modified PLA was synthesized through the reaction of collagen with acylchlorided PLA, which was prepared by acylchloriding the carboxylic groups at the open end of PLA. FTIR, FITC-labeled fluorescence spectra, XPS, and DSC thermal analysis confirmed that collagen had been successfully grafted on the PLA. Given the introduction of collagen, the hydrophilicity of collagen-PLA was better than that of PLA, and the cell compatibility of collagen-PLA with mouse embryonic fibroblasts (3T3) was significantly better than PLA in the respects of cell morphology and cell proliferation. The degradation rate of collagen-PLA was more constant than that of PLA because of the graft of collagen. This study results showed that the cell compatibility and degradability of PLA was improved by the grafting method. Moreover, collagen-PLA is a promising candidate for biomedical applications.
